# Lack of Influence of Serum Magnesium Levels on Overall Mortality and Cardiovascular Outcomes in Patients with Advanced Chronic Kidney Disease

**DOI:** 10.5402/2013/191786

**Published:** 2013-06-19

**Authors:** Olimpia Ortega, Isabel Rodriguez, Gabriela Cobo, Julie Hinostroza, Paloma Gallar, Carmen Mon, Milagros Ortiz, Juan Carlos Herrero, Cristina Di Gioia, Aniana Oliet, Ana Vigil

**Affiliations:** Nephrology Service, Hospital Severo Ochoa, Avenida Orellana s/n, Leganés 28911, Madrid, Spain

## Abstract

*Background*. Low serum magnesium has been associated with an increased cardiovascular risk in the general population and in dialysis patients. Our aim was to analyze the influence of serum magnesium on overall mortality and cardiovascular outcomes in patients with advanced CKD not yet on dialysis. *Methods*. Seventy patients with CKD stages 4 and 5 were included. After a single measurement of s-magnesium, patients were followed a mean of 11 months. Primary end-point was death of any cause, and secondary end-point was the occurrence of fatal or nonfatal CV events. *Results*. Basal s-magnesium was within normal range (2.1 ± 0.3 mg/dL), was lower in men (*P* = 0.008) and in diabetic patients (*P* = 0.02), and was not different (*P* = 0.2) between patients with and without cardiopathy. Magnesium did not correlate with PTH, calcium, phosphate, albumin, inflammatory parameters (CRP), and cardiac (NT-proBNP) biomarkers but correlated inversely (*r* = −0.23; *P* = 0.052) with the daily dose of loop diuretics. In univariate and multivariate Cox proportional hazard models, magnesium was not an independent predictor for overall mortality or CV events. *Conclusions*. Our results do not support that serum magnesium can be an independent predictor for overall mortality or future cardiovascular events among patients with advanced CKD not yet on dialysis.

## 1. Introduction

Magnesium is predominantly an intracellular cation. Serum magnesium concentration does not reflect total body magnesium content since 60% is found in the skeleton, 39% intracellular and only 1% extracellular [1].

Magnesium (Mg) plays an important role in the regulation of vascular tone and heart rhythm [2, 3]. Magnesium deficiency has been reported to promote inflammation, and it decreases the specific immune response [4]. Magnesium also reduces total peripheral resistance by stimulation of nitric oxide synthesis [5] and is a potent inhibitor of vascular calcification [6–8].

In the general population, it seems that hypomagnesaemia may play a significant role in the development of cardiovascular disease [9, 10].

The gastrointestinal tract, the skeleton, and the kidneys are integrally involved in normal magnesium homeostasis. Renal failure is the most common cause of hypermagnesemia, which is usually mild and asymptomatic. In CKD, when GFR falls to below 30 mL/min, urinary Mg excretion may be insufficient to balance intestinal Mg absorption leading to chronic Mg overload [11]. However, some conditions can lead to negative Mg balance even in these patients, such as excessive intake of diuretics, reduced gastrointestinal intake, and a low Mg concentration of dialysate [12, 13].

Cardiovascular disease is the leading cause of mortality and morbidity in patients with CKD. Several traditional and nontraditional risk factors have been identified as risk factors for the increased mortality of end-stage renal disease patients. Accelerated atherosclerosis associated with vascular calcification of intima and media layers and arterial stiffening is a frequent finding in these patients [14] and is a strong risk factor for increased morbidity and mortality [15, 16]. In hemodialysis patients, an inverse association between serum Mg and the common carotid intima-media thickness has been observed [17, 18], and some recent observational studies have confirmed the superior survival of dialysis patients with serum Mg levels above the normal range [19]. This survival advantage could be related to the inhibition of vascular calcification, phosphate-lowering effect, and to the reduction of the oxidative stress [20–22].

The aim of our study was to evaluate whether serum Mg level could be an independent predictor of mortality and future cardiovascular events even in patients with advanced renal failure not yet on dialysis.

## 2. Patients and Methods

All of the seventy patients with stages 4 and 5 CKD followed in our predialysis out-patient unit were included in the study. Mean age was 64 ± 13 years (32–87 years), and 46 patients (66%) were men. Median follow-up in our predialysis unit prior the inclusion in the study was 2 years (interquartile range: 0.9–3 years). Clearance of creatinine (CrC) was calculated using the 24-hour collection method. Mean CrC at the start was 20 ± 7 mL/min. No patient received magnesium supplements during the study.

After a single measurement of serum Mg level, patients were followed-up for time-to-event analysis until the occurrence of death or cardiovascular event. Primary end-point was all-cause mortality, and secondary end-point was fatal or nonfatal CV events. Cardiovascular events were defined as myocardial infarction, angina, sudden death of cardiac origin, heart failure episode, stroke, or complicated peripheral vascular disease. Patients who initiate dialysis during the follow-up period were followed until death, occurrence of CV events, or until the end of the study and were not censored at initiation of renal replacement therapy.

All blood samples were obtained from patients in the morning after 12 hours of fasting for measurement of the biochemical data. Besides Mg levels, biochemical data recorded included serum calcium, phosphate, alkaline phosphatase, PTH, levels of OH-vitamin D, blood Hb, serum albumin, transferrin, cholesterol, 24-hours proteinuria, CRP levels, and levels of NT-proBNP.

Serum CRP was measured by nephelometry on a BNA II (Dada Behring, Liederbach, Germany). NT-pro BNP was determined using chemiluminiscent Elecsys proBNP sandwich immunoassay (ECLIA) on an Elecsys 2010 (Roche Diagnostic, Mannheim, Germany). The levels of 25-OH-vitamin D were determined by radioimmunoassay (Dia Sorin, Stillwater, Minn, USA).

The use of erythropoiesis stimulating agents and the weekly dose employed were included in the study as well as the use of vitamin D supplements. The dose of loop diuretics was also recorded, as most of our predialysis patients used diuretics as part of our strategy of strict volume control in combination with a low sodium diet prescription. 

All the statistical analyses were performed by using SPSS 11.0 (SPSS Inc., Chicago, Ill, USA) statistical package. Nonnormally distributed variables were expressed as median (range), and normally distributed variables were as mean ± SD, as appropriate. A “*P*” value <0.05 was considered to be statistically significant. Pearson or Spearman coefficients were used to determine correlations between continuous variables. Survival and time-to-event analysis were done using the Cox proportional hazards model, including adjustment for potential confounding factors. Data are presented in the form of hazard ratios (HR) and 95% CI.

## 3. Results

Demographic characteristics of the population studied are expressed in Table 1. As the population includes relatively old patients, vascular and diabetic nephropathy are the most frequent causes of CKD. The 47 patients with CKD stage 4 had a mean CrC of 24 ± 5 mL/min whereas mean CrC in patients with CKD stage 5 was 12 ± 4 mL/min. The prevalence of cardiovascular disease was elevated in the population studied. Thirty-two percent of the patients had associated cardiopathy, defined as presence of valvular cardiopathy, hypertrophic cardiopathy, previous heart failure episodes, arrhythmias, or ischemic cardiopathy. Ischemic cardiopathy was present in 17 patients representing the most frequent cause of associated cardiopathy. Peripheral vascular disease was also frequent in this population (14%).

Table 2 expresses the biochemical parameters at baseline. Mean serum Mg level was within normal range in our patients (2.1 ± 0.4 mg/dL; normal range: 1.58–2.55 mg/dL) without significant differences between patients with CKD stages 4 and 5 (2.1 ± 0.3 mg/dL in stage 4 versus 2.2 ± 0.3 mg/dL in stage 5; *P* = 0.25).

Serum Mg was significantly lower in men (2.04 ± 0.3 mg/dL versus 2.26 ± 0.3 mg/dL in women; *P* = 0.008) and in diabetic patients (1.98 ± 0.3 mg/dL versus 2.2 ± 0.3 in non-diabetic patients; *P* = 0.02). We found no significant differences in serum Mg levels between patients with and without clinical evident cardiopathy (*P* = 0.22). Patients with and without peripheral vascular disease showed also no statistically different Mg levels (*P* = 0.48).

At baseline, serum Mg did not correlate with patient's age (*P* = 0.35). We found no significant correlation between serum Mg and bone and mineral metabolism parameters; there was no significant relationship between serum Mg and serum calcium (*P* = 0.76), phosphate (*P* = 0.65), alkaline phosphatase (*P* = 0.8), PTH (*P* = 0.4), or 25 OH vitamin D levels (*P* = 0.14). There were also no differences in Mg levels between patients with or without vitamin D supplements (2.1 ± 0.8 mg/dL in patients with vitamin D supplements versus 2.2 ± 0.3 mg/dL in patients without vitamin D; *P* = 0.25).

Serum Mg did not correlate either with nutritional parameters at baseline; there was no significant correlation with serum albumin (*P* = 0.53), transferrin (*P* = 0.21), or cholesterol (*P* = 0.51). The relationship with cardiac and inflammatory parameters was also not significant (*P* = 0.7 with NT-proBNP; *P* = 0.16 with CRP levels). We found no significant correlation between serum Mg and blood hemoglobin (*P* = 0.4) as well as with the weekly dose of erythropoietin (*P* = 0.94). On the contrary, serum Mg correlated inversely with the daily dose of loop diuretics, although the correlation did not achieve statistical significance (*r* = −0.23; *P* = 0.052).

During the follow-up period, 6 patients (9%) died, in 11 patients (17%) a fatal or nonfatal CV event occurred, and 13 patients (20%) started renal replacement therapy. These patients were followed until death, the development of CV event, or until the end of the study; thus, patients who started dialysis were not censored at the initiation of dialysis.

**Figure 1:**
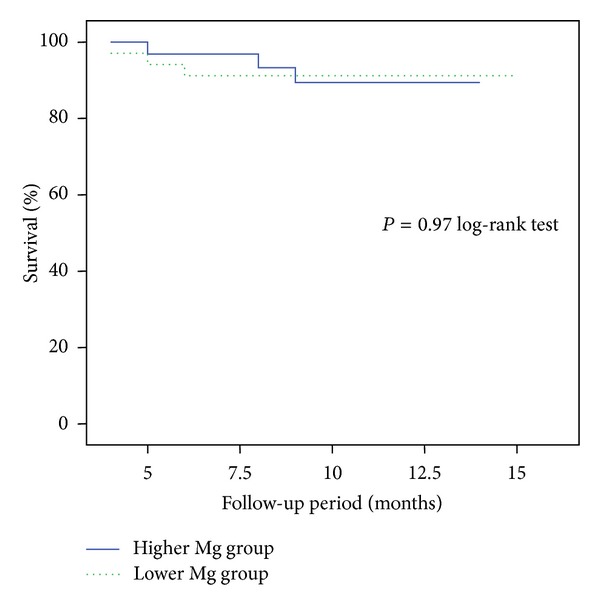
Kaplan-Meier survival curves according to median serum magnesium levels (<2.1 mg/dL or ≥2.1 mg/dL).

**Figure 2:**
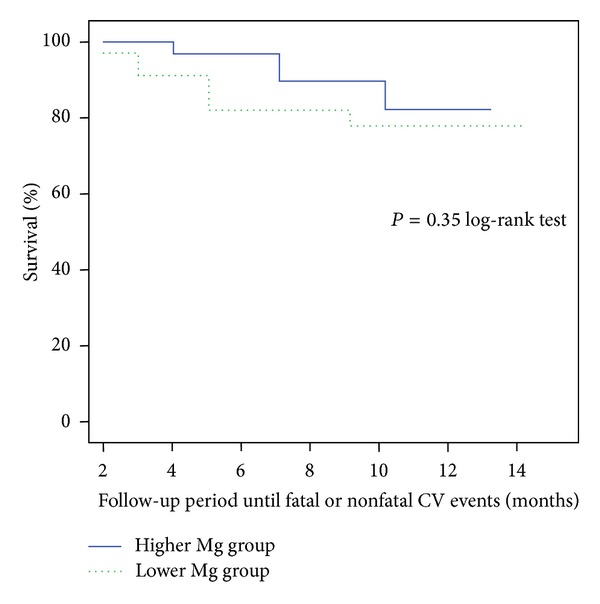
Kaplan-Meier development of fatal and nonfatal cardiovascular events curves according to median serum magnesium levels (<2.1 mg/dL or ≥2.1 mg/dL).

The mean follow-up period was 11 ± 2.8 (range 4–15) months. Primary end-point was all-cause mortality, and secondary end-point was the development of fatal or nonfatal CV event. The predictors for time to-death and time to-CV events were studied by univariate and multivariate Cox regression analysis. Serum Mg does not predict death for all causes and was not an independent predictor of fatal or nonfatal CV event neither in univariate nor in multivariate analysis.  Figure 1 shows the Kaplan-Meyer curves of all-cause mortality according to median serum magnesium levels (<2.1 mg/dL or ≥2.1 mg/dL); there were no significant differences in both survival curves (*P* = 0.97 by the log-rank test).  Figure 2 expresses the development of fatal or nonfatal CV event according to median magnesium levels; there was also no significant differences between the two curves (*P* = 0.35 by the log-rank test).

In univariate Cox regression, patient's age, the presence of peripheral vascular disease, serum albumin, and C-reactive protein and creatinine clearance were independent predictors of all-cause mortality (Table 3). In a forward conditional multivariate Cox regression analysis, the factors which significantly contributed to predicting outcome independent of each other and confounders were CRP (HR 1.1; CI: 1.03–1.12; *P* = 0.04), the presence of peripheral vascular disease (HR 18.6; CI 1.9–180; *P* = 0.01), and serum albumin (HR 0.06; CI 0.005–0.77; *P* = 0.03). Of note is that serum magnesium does not influence on all-cause mortality either in univariate or in multivariate Cox regression analysis.

The independent predictors of the development of fatal or nonfatal CV events in univariate analysis are expressed in Table 4. Previous history of cardiopathy, peripheral vascular disease, diabetes, serum albumin and transferrin were independent predictors of future CV events. The influence of 24-hour proteinuria was in the limit of statistical signification (*P* = 0.06) in univariate analysis. The multivariate stepwise forward conditional Cox regression analysis shows that only peripheral vascular disease (HR 22.7; CI 2.7–190; *P* < 0.005) and 24-hour proteinuria (HR 1.5; CI 1.12–2.2; *P* < 0.01) are independent predictors of CV events after adjustment for confounders. Newly, serum magnesium did not influence on CV events neither in univariate nor in multivariate Cox regression analysis.

## 4. Discussion

The main finding of our study is that serum magnesium level does not seem to be an independent predictor of overall mortality or future cardiovascular events among patients with advanced CKD not yet on dialysis, at least at short-term followup. Serum magnesium level also does not correlate with bone and mineral metabolism parameters in our predialysis patients.

Our results are not consistent with those observed by other authors who suggest a survival advantage in patients with slightly elevated serum magnesium concentration. In hemodialysis patients, some authors [19] have found that mortality rates were significantly higher in the group with lower baseline magnesium levels and that serum magnesium levels were a significant and independent predictor of overall mortality but not for death from cardiovascular causes. More recently, Kanbay and colleagues [22] evaluated the possible contribution of magnesium on cardiovascular outcome in patients with moderate-to-severe CKD not yet on dialysis. They found that serum magnesium may be an independent predictor of future cardiovascular outcomes. Compared to our study, Kanbay and colleagues included patients with a wider range of creatinine clearance as there were a high proportion of patients with stage 3 CKD, no patients received diuretics, and finally mean follow-up period was longer than in our study. Unlike the Kanbay's study, most of our predialysis patients used loop diuretics at different doses as part of our strategy of strict volume control, which consists of a prescription of low sodium diet and the rational use of loop diuretics in order to achieve adequate blood pressure and volume control. Furthermore, we found an inverse correlation in the limit of significance between serum magnesium and the daily dose of loop diuretics. Thus, we could hypothesize that serum magnesium could be modified by the use of diuretics, and this could explain the lack of influence of serum magnesium levels on cardiovascular outcomes in our patients. Otherwise, as our follow-up period was shorter than in the Kanbay's study, we cannot rule out that magnesium concentration could have any influence on outcomes at longer follow-up periods.

According to other studies [23], serum magnesium levels were lower in our diabetic patients. Surprisingly, we did not find any relationship between serum magnesium and bone and mineral metabolism parameters. In vitro and in vivo studies have demonstrated that magnesium concentrations modulate PTH secretion in a similar manner to calcium [24, 25], and several authors have observed an inverse correlation between serum magnesium and PTH levels in dialysis patients [26, 27]. However, other studies failed to show a correlation between serum magnesium and serum PTH levels in peritoneal dialysis patients [28]. Otherwise, many of the studies evaluating the association between PTH and magnesium are not controlled well enough or suffer from other methodological drawbacks to draw firm conclusions [11]. Independent of its effect on PTH levels, magnesium is a potent inhibitor of vascular calcification via multiple molecular mechanisms [6–8, 11]. Unfortunately, data of vascular calcification were not analyzed in our patients. Magnesium also participates in the atherosclerotic process by its effect on endothelial function. It has been suggested that high magnesium level can improve endothelial function via its anti-inflammatory effect. This hypothesis is supported by recent studies in which magnesium intake was inversely associated with C-reactive protein concentration [29]. However, we did not find any significant relationship between serum magnesium and CRP levels in our patients. Serum magnesium also did not correlate with biochemical markers of cardiac disease such as NT-proBNP levels, and serum magnesium was not different in patients with or without cardiac disease at baseline.

## 5. Conclusions

Contrary to previous reports in the general population and in patients with CKD, the present study does not support that low serum magnesium level could influence on overall mortality or future cardiovascular events among patients with advanced CKD not yet on dialysis at short-term (one year) follow-up. As most of our patients used loop diuretics and an inverse correlation between serum magnesium and the daily dose of loop diuretics was found in our study, we could hypothesize that serum magnesium could be modified by the use of diuretics, and this could explain the lack of influence of serum magnesium levels on cardiovascular outcomes in our patients. 

The small sample size and the relative short follow-up time of our study could have biased our findings. Thus, further studies are needed to confirm our results or, on the contrary, to support the results of other studies that suggest a detrimental effect of low serum magnesium on mortality or cardiovascular outcome in this population.

## Figures and Tables

**Table 1:** Demographic characteristics of the population studied.

Age (years)	64 ± 13*
Sex (men)	46 (66)
Causes of CKD	
Diabetes	23 (34)
Vascular	18 (26)
Chronic glomerulonephritis	16 (23)
Interstitial	4 (6)
PQKD	2 (3)
Unknown	4 (6)
Other nephropaties	1 (2)
CKD stages	
Stage 4 (%)	47 (67)
Stage 5 (%)	23 (33)
Vintage in predialysis (years)	2 (0.9–3)^†^
Associated cardiopathy	22 (32)
Peripheral vascular disease	10 (14)

Data expressed as number (percentage), *mean ± SD or ^†^median (interquartile range). CKD: chronic kidney disease; PQKD: polycystic kidney disease.

**Table 2:** Biochemical data at baseline of the population studied.

CrC (mL/min)	20 ± 7
Magnesium (mg/dL)	2.1 ± 0.4
Calcium (mg/dL)	9.3 ± 0.6
Phosphate (mg/dL)	4.2 ± 0.9
Alkaline phosphatase (IU/L)	96 ± 38
PTH (pg/mL)	302 ± 239
25 OH vitamin D (ng/mL)	16 ± 11
Albumin (g/dL)	4.1 ± 0.4
Transferrin (mg/dL)	214 ± 38
Cholesterol (mg/dL)	176 ± 39
Proteinuria (g/24 h)	0.8 (0.4–2.2)
Hemoglobin (g/dL)	12.6 ± 1.6
CRP (mg/L)	4 (1.8–11)
NT-proBNP (pg/mL)	626 (251–1567)

Data expressed as mean ± SD or median (interquartile range).

CrC: clearance of creatinine; PTH: parathyroid hormone; CRP: C-reactive protein.

**Table 3:** Independent predictors of overall mortality. Univariate Cox analysis.

	HR	95% CI	*P *
Age (years)	1.13	1.02–1.24	**0.021**
Diabetes (yes/no)	3.71	0.7–20.6	n.s
Cardiopathy (yes/no)	1.6	0.27–9.6	n.s.
Peripheral vasc. dis. (yes/no)	14.1	1.6–121	**0.016**

Mg (mg/dL)	1.5	0.15–14.7	n.s
Calcium (mg/dL)	0.4	0.12–1.5	n.s
Phosphate (mg/dL)	1.04	0.43–2.5	n.s
PTH (pg/mL)	0.99	0.99–1.003	n.s
25 OH vitamin D (ng/mL)	0.79	0.59–1.06	n.s

Albumin (g/dL)	0.098	0.02–0.44	**0.002**
Transferrin (mg/dL)	0.98	0.96–1.003	n.s
Proteinuria (g/24 h)	1.07	0.79–1.01	n.s
CrC (mL/min)	0.8	0.7–0.97	**0.02**
Hemoglobin (g/dL)	0.59	0.34–1.05	n.s
CRP (mg/L)	1.1	1.03–1.18	**0.03**

**Table 4:** Independent predictors of fatal and non fatal cardiovascular events. Univariate Cox analysis.

	HR	95% CI	*P *
Age (years)	1.03	0.99–1.08	n.s
Diabetes (yes/no)	10.05	2.1–46.9	**0.003**
Cardiopathy (yes/no)	3.7	1.05–13.4	**0.04**
Peripheral vasc. dis. (yes/no)	9.7	2.5–37.4	**0.001**

Mg (mg/dL)	0.4	0.08–2.5	n.s.
Calcium (mg/dL)	0.8	0.31–2.16	n.s.
Phosphate (mg/dL)	1.26	0.72–2.2	n.s.
PTH (pg/mL)	1.001	0.99–1.002	n.s.
25 OH vitamin D (ng/mL)	0.76	0.22–2.6	n.s.

Albumin (g/dL)	0.12	0.04–0.39	**0.001**
Transferrin (mg/dL)	0.98	0.97–0.99	**0.03**
Proteinuria (g/24 h)	1.17	0.99–1.01	0.06
CrC (mL/min)	0.92	0.85–1.008	n.s.
Hemoglobin (g/dL)	0.79	0.53–1.19	n.s.
CRP (mg/L)	1.03	0.97–1.09	n.s.
